# Craniotomy, a Case of

**Published:** 1886-10

**Authors:** Peyton Turner

**Affiliations:** Abilene, Texas


### CRANIOTOMY.

### Attempt at Indictment of the Attending Physician.

*By Peyton Turner, M. D.*

[For Daniel's Texas Medical Journal.]

MRS B., aged 20, primipara, began to have occasional slight pains on the 9th of November last. The case had advanced to term, and on the 10th the contractions of uterus increased in frequency and severity, denoting the advent of labor nigh at hand. At 12 m. she took to bed, and touch revealed a slightly dilated os. Not wishing to interfere with the uterine contractions, and hoping to diminish the acuteness of the pains, gave chloral, and noted the dilation as it proceeded with great rapidity. The head could but partially engage after complete dilation. and, as the pains now were excruciating and frequent, I came to the conclusion that unaided nature was inadequate to effect delivery. Conjoined manipulation revealed an enormous head, but I was at a loss to know whether to suspect over-development or intra-uterine hydrocephalus. Regarding as most conservative, to first attempt delivery with the forceps, I made use of the Simpson long forceps, but in this I was unsuccessful. I have delivered with forceps a number of times, and this was the first time failure attended my efforts. After fruitless efforts to engage the head, I withdrew the blades, and concluded to wait a little while longer on nature. The pain became almost continuous, and of such severity that I feared a rupture of the uterus would take place. Signs of exhaustion began to show themselves, and now relief was imperative, or my patient would perish. Still deferring the operation of craniotomy, I made another attempt at instrumental delivery. This time I was successful in engaging the head, but the disproportion was such that I was soon convinced that diminution of the size of the head offered the only chance of safety to the mother. Something must be done, and the patient's condition, by this time, suggested that any further delay would probably prove disastrous. I told the family it was possible to save the life of the mother by sacrificing the child; consent being obtained, the cranium was punctured, and a gush of hydrocephalic fluid took place; then I made traction by the forceps (which had not been removed,) and brought the head down to the inferior strait. The head was not sufficiently reduced, however, to be accommodated at the inferior strait, and required an incision through which I removed the cranial contents, then compressed the head and completed delivery by the forceps at about 5 o'clock. The placenta was forced into the vagina in due time and was removed with the hand; I also administered ergot. I have failed to mention the use of chloroform from time to time, as the case demanded. Antiseptic injections were used twice daily for eight or ten days, and convalescence was rapid so far as parturition was concerned.

The second night following labor, the patient was seized with acute dysentery, and doubting the propriety of beginning the treatment with purgatives, I at once resorted to astringents. This complication resisted such remedies as Dover's powder, opium, ipecacuanha, carbolic acid, kino, plumbic acetate, ferric and cupric sulphates, etc. A strict milk diet only was allowed, but the disease ran its course.

Certain parties inimical to my welfare sought to have me indicted; and the grand jury were, from duty, bound to investigate the case. They did not examine all the witnesses, however, presuming, no doubt, that it would be useless to try further to find a bill of indictment against me. The case no doubt had been grossly misrepresented.

As to the justifiability of the operation, let us refer to Playfair's Obstetrics a moment and see:

Page 498—" Want of proportion between the fœtus and the pelvis, depending on undue size of the head, either natural or the result of disease, may render the operation essential. In the former of these cases, we shall generally have first attempted delivery with the forceps, and, if it has failed, there can be no doubt as to the propriety of lessening the bulk of the head by perforation."

If an aperture one inch in diameter would admit the passage of a solid body two inches in diameter, then it would be possible to deliver in intro-uterine hydrocephalic cases without any great regard for mechanical rules or physical laws. Such, however, would be contrary to reason, and common sense would suggest a diminution of the size of the solid, or an enlargement of the aperture.

In speaking of the treatment of these hydrocephalic cases, Playfair says it "consists in tapping the head, so as to allow the cranial bones to collapse."

We would infer from the records of Celsus, Ætius and Hippocrates, that the operation was not unknown to them, and since man first began to write up human ailments, it is pretty certain that there is not a civilized race on the globe who do not regard the life of the mother as paramount to that of the fœtus, and I think the day has already come, (and been with us some time, as to that,) when a man who would set with folded hands and let the parent perish, should be held criminally responsible for the same, as it is certain both mother and child would perish under the circumstances. Statistics show that rupture of the uterus is quite likely to occur in cases of hydrocephalus, and this, of itself, is a sufficient guarantee that a very early interference is necessary, and if, from physical signs, we conclude the child already dead, then I would advise the interference almost at the inception of labor, or as soon as sufficient dilation had taken place, and by so doing, save precious lives; notwithstanding we may run the risks of being arraigned before a tribunal of justice, through malevolence of personal enemies, who are on the lookout for some way to injure those who succced where they fail.

Abilene, Texas, August 6, 1886.

